# Benefit of Oleuropein Aglycone for Alzheimer's Disease by Promoting Autophagy

**DOI:** 10.1155/2018/5010741

**Published:** 2018-02-20

**Authors:** Joaquín G. Cordero, Ramón García-Escudero, Jesús Avila, Ricardo Gargini, Vega García-Escudero

**Affiliations:** ^1^Departamento de Anatomía, Histología y Neurociencia, Facultad de Medicina, UAM, Arzobispo Morcillo 4, 28029 Madrid, Spain; ^2^Molecular Oncology Unit, CIEMAT, Complutense 40, 28040 Madrid, Spain; ^3^Biomedical Research Institute I+12, University Hospital 12 de Octubre, Avenida de Córdoba s/n, 28041 Madrid, Spain; ^4^Centro de Investigación Biomédica en Red de Cáncer (CIBERONC), Madrid, Spain; ^5^Centro de Biología Molecular “Severo Ochoa” (UAM-CSIC), Nicolás Cabrera 1, Cantoblanco, 28049 Madrid, Spain; ^6^Centro de Investigación Biomédica en Red de Enfermedades Neurodegenerativas (CIBERNED), Valderrebollo 5, 28031 Madrid, Spain; ^7^Neuro-oncology Unit, Instituto de Salud Carlos III-UFIEC, Crtra, Pozuelo Km 2, Majadahonda, 28220 Madrid, Spain

## Abstract

Alzheimer's disease is a proteinopathy characterized by accumulation of hyperphosphorylated Tau and *β*-amyloid. Autophagy is a physiological process by which aggregated proteins and damaged organelles are eliminated through lysosomal digestion. Autophagy deficiency has been demonstrated in Alzheimer's patients impairing effective elimination of aggregates and damaged mitochondria, leading to their accumulation, increasing their toxicity and oxidative stress. In the present study, we demonstrated by microarray analysis the downregulation of fundamental autophagy and mitophagy pathways in Alzheimer's patients. The benefits of the Mediterranean diet on Alzheimer's disease and cognitive impairment are well known, attributing this effect to several polyphenols, such as oleuropein aglycone (OLE), present in extra virgin olive oil. OLE is able to induce autophagy, achieving a decrease of aggregated proteins and a reduction of cognitive impairment in vivo. This effect is caused by the modulation of several pathways including the AMPK/mTOR axis and the activation of autophagy gene expression mediated by sirtuins and histone acetylation or EB transcription factor. We propose that supplementation of diet with extra virgin olive oil might have potential benefits for Alzheimer's patients by the induction of autophagy by OLE.

## 1. Introduction

### 1.1. Alzheimer's Disease

Alzheimer's disease (AD) is a progressive, fatal, and currently incurable neurodegenerative disease. It is clinically characterized by a gradual loss of cognitive function, including slow deterioration of memory, reasoning, abstraction, language, and emotional stability [1]. As a consequence, in the final stages of the disease, the patient is unable to perform any daily task without adequate assistance from family members or social services [1]. AD is the most common cause of dementia worldwide, accounting for between 50% and 70% of the cases recorded among people over 65 years old. The aging population presents the highest risk of the disease, especially in developed countries, therefore the number of affected people is expected to increase dramatically up to 115 million in 2050 [2].

The causes of the disease have not been fully clarified, but several risk factors have been associated with AD. These are genetic factors (presenilin 1 and 2, apolipoprotein E*ɛ*4 allele), vascular events, history of traumatic brain injury, oxidative stress, decreased endothelial nitric oxide production and consequent inflammation, hypertension, hyperhomocysteinemia, diabetes, insulin resistance, hypercholesterolemia, obesity, hormonal alterations, lifestyle factors (saturated fat intake, vitamin E intake, low physical activity, smoking, etc.), and psychological factors [3].

The main histologic sign confirming the AD diagnosis is the presence of intracellular neurofibrillary tangles of hyperphosphorylated Tau protein and extracellular deposits of beta-amyloid (A*β*) peptide (senile plaques) in certain areas of the brain. Tau belongs to the microtubule-associated proteins (MAPs) family that participates in the assembly and stabilization of microtubules. This process is necessary for the maintenance of cellular shape and transport of proteins, organelles, and other biological components through axons [4]. Additionally, it is important in the connections between microtubules and other elements of the cytoskeleton such as neurofilaments, spectrin, or actin filaments [4]. The A*β* is a peptide that contains between 39 and 43 amino acids, being a natural product of proteolytic processing of the amyloid precursor protein (APP), due to the sequential action of enzymes *β*- and *γ*-secretase [5].

For causes not entirely clarified, in AD, both proteins tend to generate cytotoxic aggregates. These trigger series of neuronal alterations such as loss of synaptic transmission, gliosis or proliferation and abnormal activation of glial cells (astrocytes and microglia) [6], vascular dysfunction due to fibrillary amyloid deposition at cerebral vessel walls [7], increased oxidative stress, augmented inflammatory response, and deregulation of calcium homeostasis [8].

### 1.2. Autophagy Pathway

Within eukaryotic cells, there are two systems responsible for the degradation of cytoplasmic proteins: the ubiquitin-proteasome system (UPS) and the autophagy. In the case of poorly folded protein aggregates, it is suggested that autophagy (specifically macroautophagy) is more efficient in degrading them than UPS. This is due to the small pore size of proteasomes, which hinders the entry of proaggregating oligomers and their subsequent elimination [9].

Autophagy is a process by which the different cellular components and organelles are transferred to lysosomes to be degraded by their hydrolytic enzymes [10]. It is a key process for the correct cell function, acting as a recycling system in energy restriction conditions and allowing the cell to degrade nonessential organelles and proteins to reuse their components. In addition, it functions as a system for eliminating aggregated protein and cytotoxic damaged organelles [11].

Macroautophagy process (or “autophagy” as we will call it from now onwards for simplicity) can be divided into three phases [11]: autophagosome formation, substrate recognition, and autophagosome trafficking and degradation.

#### 1.2.1. Formation of the Autophagosome

First, cellular materials to be degraded are isolated into a double membrane vesicle known as autophagosome. Its formation requires the fusion of several smaller membranous vesicles to form a flattened structure called phagophore. This fusion process continues until the autophagosome with a double membrane is formed enveloping small fragments of the cytoplasm and organelles. Initiation of phagosome formation requires activation of the quaternary complex ULK1-ATG13-ATG101-FIP200 [12]. This complex is controlled by two signaling pathways: the *mTORC1 pathway* which acts as an autophagy blocker by inhibiting ULK1 phosphorylation and the *adenosine monophosphate activated protein kinase* (AMPK) which activates ULK1 by phosphorylation of mTORC1 different residues [13]. Once activated ULK1 phosphorylates and activates Beclin-1, which in turn triggers the activation of the VPS34 complex. The latter produces an accumulation of phosphatidylinositol-3-phosphate (PI3P) in phagophore, allowing the recruitment of numerous binding proteins among which the ATG5-ATG12-ATG16L1 complex stands out. This complex is necessary for phagophore elongation through the binding to microtubule-associated protein 1 light chain 3 (LC3), which after several conjugations will result in the active form LC3-II [14]. Finally, the ATG5-ATG12-ATG16L1 complex is dissociated from the phagophore membrane and it ends up closing, creating the autophagosome and leaving LC3-II associated with the two faces (external and internal) of autophagosomes [14].

#### 1.2.2. Substrate Recognition and Selective Autophagy

There are several adaptor proteins that recognize cargo to be engulfed into autophagosomes such as p62 (sequestosome 1), Next to BRCA1 gene 1 protein (NBR1), Nuclear Domain 10 Protein 52 (NDP52; also known as CALCOCO2), and optineurin. These proteins recognize substrates specifically labeled for degradation by ubiquitination, for example, and bind to LC3 family proteins of phagosome mediating the recruitment of cargoes [15]. Selective targeting can also be mediated by autophagy receptors that form a bridge between the cargo-autophagy receptor complex and components of the autophagosome membrane such as ATG5 and PI3P [16]. This is the case of autophagy-linked FYVE protein (ALFY) that serves as a scaffold protein for p62 mediated labeling [16]. Additionally, BNIP3L is a mitochondrial membrane protein containing a LC3-interacting region motif involved in the targeted clearance of damaged mitochondria [17].

#### 1.2.3. Autophagosome Trafficking and Degradation

Once the autophagosome is formed, it is transported by dynein engines through microtubules to the perinuclear region. In this pathway, autophagosome may fuse with vesicles from the endocytic pathway to form amphysomes. Finally, autophagosomes and amphysomes fuse with lysosomes forming autolysosomes [18]. In them, the degradation of the compounds will be achieved, thanks to the combined action of acid pH and lysosomal enzymes. The macromolecules resulting from lysosomal digestion are released to the cytosol by permeases [19]. Transcription factor EB (TFEB) is as a master regulator for lysosomal biogenesis, therefore its activation favors the degradation phase of autophagy [20].

### 1.3. Autophagy Deficiency in AD

The accumulation of extracellular protein aggregates, mainly composed by polymeric A*β*42 peptide, a product of proteolysis of APP, is one of the main responsible for neurological damage and cognitive deficit. Under normal conditions, protein aggregates and damaged organelles are eliminated through autophagy system, avoiding their cytotoxic effect. But, as a consequence of age and oxidative stress, the efficacy of this system is reduced. This results in the accumulation of poorly digested proteins in autophagic vacuoles and damaged mitochondria, which cause an increase in oxidative stress and neuronal death [21].

Several key regulatory proteins in autophagy are reduced in AD, such as Beclin-1, PARK2/parkin and Nuclear Receptor Binding Factor 2 (NRBF2). Beclin-1 is a fundamental protein for autophagy regulation and cell death that is found decreased in brain samples from early-stage Alzheimer's patients [22]. In addition, inhibition of Beclin-1 gene expression in a mice model that expresses human APP produces intraneuronal accumulation of A*β*, extracellular A*β* deposition, and neurodegeneration [22]. PARK2 is one of the proteins involved in mitophagy, a specialized form of autophagy by which mitochondria are selectively degraded and recycled. PARK2 labels damaged mitochondria for their subsequent degradation by its E3 ubiquitin ligase activity. PARK2 is found reduced in the cerebral cortex of AD patients, leading to a pathological increase of oxidative stress [23]. NRBF2 is a Beclin-1-Vps34-binding protein that modulates autophagy via Atg14L-linked Vps34 activity regulation [24]. NRBF2 expression is found reduced in the hippocampus of transgenic mice model that reproduces amyloid pathology characteristic of AD in humans [25].

It has been also proposed that A*β* can cause a pathological accumulation of autophagic vacuoles. Intracellular A*β* can also modulate the autophagy process by RAGE-calcium-CaMKK*β*-AMPK pathway [26] or by generating ROS by mitochondrial damage [27].

## 2. Gene Expression Profiling Demonstrates Autophagy Dysfunction in AD

**Figure 1:**
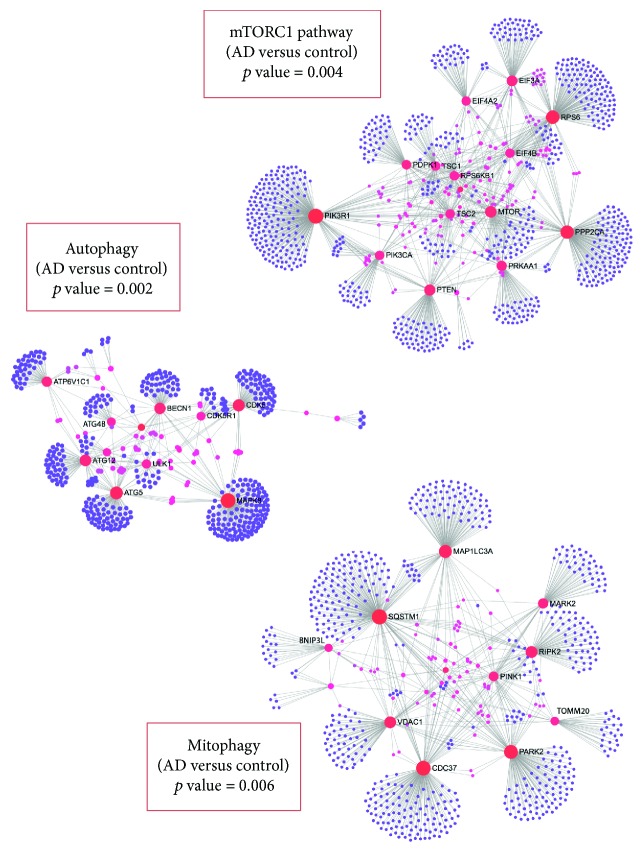
Gene expression profiling revealed dysregulation of mTOR, autophagy, and mitophagy pathways in Alzheimer's disease patients. Genes having significant differential expression between brain samples from normal controls subjects and AD patients were extracted. The analysis was done from a microarray dataset having 253 samples from 84 patients (*n* = 56 normal; *n* = 28 AD patients [30]). Samples were collected from four brain regions: hippocampus, entorhinal cortex, superior frontal cortex, postcentral gyrus. Enrichment in pathways and biological processes of deregulated genes was done using GSEA and Gene Ontology analyses, and the significance of the results (*p* value) for each pathway was represented in the graph. We could observe that mTOR pathway was significantly upregulated, whereas autophagy and mitophagy were significantly downregulated in AD. Genes that exhibited up or downregulation with *p* value <0.01 using two-tailed Student's *t*-test were selected for representation. Graphs show nodes plots of these altered genes in AD versus controls in each of these pathways using NetworkAnalyst software (http://www.networkanalyst.ca/faces/home.xhtml).

In the last years, the advances of high-throughput genomic analysis have generated data from patients that allow us the integration of gene deregulations and the determination of what biological processes are affected in different pathologies such as neurodegenerations. Particularly in AD, these gene expression datasets have demonstrated a significant deregulation of biological processes associated with calcium signaling, inflammation, and mitochondrial dysfunction [28]. Several of these processes are known to have a deep implication in AD pathology. However, these are not related to one of the main defects associated to AD, as it is proteotoxicity [28, 29]. Trying to shed light on this issue, we have analyzed a microarray dataset of brain samples of AD patients compared to healthy subjects, in which we focused in protein degradation-associated processes. The row data of this microarray dataset were previously published by the group of Berchtold [30]. Using gene enrichment analysis of main genes down or upregulated in AD versus healthy with significant differences (*p* value <0.01), we analyzed what biological processes were altered by Gene Ontology. We could observe that several metabolism and catabolism linked processes such as mTORC1 pathway, autophagy, and mitophagy were affected in AD with respect to healthy individuals. These results are shown in Figure 1, in which the principal modified genes were represented as well as the gene enrichment *p* values for each pathway. Multiple genes from mTORC1 pathway were increased in AD patients which generates an inhibitory effect over autophagy route, as we mentioned before. Accordingly, autophagy and mitophagy pathways were downregulated in these patients' brain samples. These effects together determine that the autophagy process was impaired in AD. Due to the fact that our data is derived from a large number of patients and different brain zones, this kind of study by global genomic analysis allows unraveling how specific biological processes with deep clinic and therapeutic involvement are affected. These analyses as well as multiple other studies that demonstrate an alteration of metabolic/catabolic pathways that converge in autophagy deficiency show us an evident necessity to evaluate new compounds that modulate these routes.

## 3. Activation of Autophagy as a Therapeutic Strategy for AD

If the autophagy pathway is altered as part of the pathological process of AD, autophagy activation may be effective in combating the cellular aggregate characteristic of this pathology. Numerous studies have shown that the pharmacological activation of autophagy might be beneficial for AD pathology. For example, the effect of 10-week treatment with rapamycin, an mTOR inhibitor, was studied in a transgenic mice model that reproduces amyloid and Tau pathology characteristic of AD in humans. They could achieve a reduction of Tau and A*β* accumulation as well as cognitive and memory improvement measured by the spatial reference version of Morris's water maze associated to increased autophagy [31]. However, in a later study, it was demonstrated that rapamycin accelerated the degeneration of motor neurons and reduced life expectancy in transgenic mice models. These results suggested that the pharmacological activation of rapamycin might affect other pathways that restricted neuron survival [32].

Other studies have demonstrated the beneficial effect of promoting autophagy by increasing PARK2 levels. These works use viral vectors to transfer PARK2 to a triple transgenic AD mice model demonstrating the enhancement of A*β* and phospho-Tau clearance by autophagy activation that improved mitochondrial function and restored synaptic function [33, 34]. This mitophagy improvement was further demonstrated in a human cell model of sporadic AD [23].

On the other hand, the activation of degradation phase of autophagy has also demonstrated its therapeutic potential in AD. The deletion of cystatin B, an endogenous inhibitor of cysteine proteases, to relieve cathepsins inhibition improving lysosomal function demonstrated to decrease extracellular amyloid pathology. This change prevented the development of learning and memory deficits of a transgenic model of AD (TgCRND8 mice that overexpress APP695 version including Swe and Ind mutations) [35].

Although the activation of autophagy has demonstrated a proven effect in the early stages and as a preventive, it is still under debate whether this may have poor effect in the advanced stages of the disease [36].

All these works are the proof of concept that activating autophagy may be used as a therapeutic approach for AD. Therefore, it is necessary to find new substances to induce autophagy minimizing collateral effects.

## 4. Neuroprotective Role of Mediterranean Diet and Extra Virgin Olive Oil

Alzheimer's disease is a multifactorial pathology in which both genetic and environmental factors are involved, highlighting among the latter certain aspects of lifestyle such as nutrition. Diet is a modifiable risk factor for dementia; thus, increasing efforts have been done to find nutrients that help to fight cognitive impairment. Some diets have been associated to reduced risk of AD; therefore, they may be helpful to find new compounds that may be beneficial for AD patients. LipiDiDiet is a research consortium, which studies the preclinical and clinical impact of nutrition in Alzheimer's disease (http://www.lipididiet.eu/). A recent study has demonstrated that the multinutrient combination Fortasyn Connect was able to slow hippocampal atrophy and functional decline by supplying rate-limiting compounds for brain phospholipid synthesis [37].

The Mediterranean diet (MD) has already demonstrated on multiple occasions its beneficial effects in preventing age-specific defects, including attenuating and preventing AD and cognitive impairment [38, 39]. For example, the 4-year study in a population of 2258 New York City residents found that increased adherence to MD reduced the risk of developing AD and was especially effective in preventing the conversion of mild cognitive impairment to AD [40]. This study showed that individuals with high adherence to MD had a 40% lower risk of developing AD and a 48% lower risk of progressing from mild cognitive impairment to AD [40]. In another study using a population of 1410 Bordeaux citizens over 65 years of age, high adherence to MD was associated with a reduction of cognitive impairment demonstrated by the Mini-Mental State Examination [41].

This neuroprotective effect has been associated with several foods found in MD, including wine and extra virgin olive oil (EVOO) standing out, the latter being one of the most internationally recognized due to its multiple beneficial properties. Among them, it is worth mentioning its role as a mental health promoter and in slowing cognitive decline in neurodegenerative diseases in the elderly population. This was demonstrated by the “three-cities study” on an elderly population of more than 8000 subjects, being the first report relating olive oil consumption with lower reduction of visual memory in a population over 65 years old [42].

Similar beneficial effect has been observed in both in vitro and in vivo studies where supplementation with EVOO is able to reduce the advance of Tau and A*β* pathology and cognitive deterioration [43–45]. The most recent study concludes that consumption of EVOO in early ages and for a long time could provide a protective effect against AD pathology and cognitive deterioration [45].

Several studies in animal models have shown that this beneficial action is due to a series of substances known as polyphenols [46], including oleuropein aglycone (OLE) present in EVOO [47].

## 5. Oleuropein Aglycone as an Autophagy Inducer

### 5.1. Polyphenols and Autophagy

Since the discovery of the benefits of moderate wine consumption for the prevention of dementia thanks to the presence of a substance called resveratrol (and not alcohol as initially suggested), numerous studies have attributed the beneficial effects of polyphenols on health. They are considered the substances responsible for the multiple mental health benefits attributed to the Mediterranean and Oriental diet [48].

Polyphenols are an extensive group of nonenergetic substances present in plant-based foods characterized by the presence of one or more phenolic rings. They can be classified according to their number of phenolic rings and the structural elements present these rings. The main groups are phenolic acids (derived from hydroxybenzoic acid or hydroxycinnamic acid), stilbenes, lignans, phenolic alcohols, and flavonoids [49].

An interesting fact showed by the different studies is that polyphenols do not act in a single pathway or modulating a certain aspect of AD, such as inhibition of A*β* aggregation. Their neuroprotective function comes from the modulation of different cellular and tissue processes that together are responsible for the reduction of cognitive impairment [35]. The A*β* toxic aggregates inhibition and the decrease in ROS produced by mitochondria and neuroinflammation are the main positive effects of polyphenols. But some of these effects may be in part due to another not so well studied action of these substances, and that is the induction of autophagy [50].

As we have previously described, autophagy plays a crucial role in AD, whose alterations have been considered preclinical events to pathology. In addition, induction of this process has proven to be a promising target for pharmacological action. Hence, it would be very helpful to look for new substances that have a positive effect on this detoxification system, and this is where polyphenols come in.

There are multiple pathways by which polyphenols can modulate autophagy, and not all of them act through the same route. See Hasima and Ozpolat [51] for a summary of involved pathways.

Polyphenols are able to modulate autophagy through canonical (Beclin-1 dependent) and noncanonical (Beclin-1 independent) signaling pathways. In the canonical pathway, Beclin-1 together with the regulatory subunits Vps34 and Vps15 form a protein complex known as Class III phosphatidylinositol 3-kinase (PI3K), inducing autophagy by modulating the autophagosomal nucleation. While in noncanonical or Beclin-1 independent, two activation mechanisms can be given, Atg5/Atg7 dependent or independent [51, 52].

For example, genistein is able to induce PI3K/Akt-dependent autophagy by reducing Akt phosphorylation levels, leading to autophagy induction as a false signal of calorie restriction [53]. Curcumin performs its function through inhibition of Akt/mTOR/p70S6K protein complex and Erk1/2 protein kinase activation, demonstrating an increase of autophagy in glioblastoma cells [54]. Rottlerin, a polyphenol extracted from the Asian *Mallotus philippensis* tree, has demonstrated its effect as an autophagy inducer through its antioxidant function. Rottlerin blocks the generation of free radicals and triggers inhibition of NF-*κ*B and activation of AMPK, which is associated with a decrease of ATP levels. The cell translates this ATP drop as a state of caloric restriction, so it activates autophagy as a regulatory mechanism [55]. Rottlerin is also able to activate autophagy through inhibition of PKC*δ*, a protein kinase that blocks autophagy through activation of tissue transglutaminase 2 (TG2) [56].

One of the most studied mechanisms of action of polyphenols is through sirtuins, a group of deacetylases that regulate cellular functions as important as metabolism, immunity, inflammation, and cell survival. Sirtuins act as modulators of autophagy both directly by promoting the deacetylation of key components such as autophagic gene products Atg5, Atg7, and Atg8 and indirectly by regulating FOXO3a transcription factor [52]. Once activated, FOXO3a induces autophagy by synthesis of glutamine synthetase, an enzyme that increases glutamine levels. This increase in glutamine causes inhibition of mTOR and the consequent activation of autophagy [57]. Resveratrol, genistein, and quercitin have been shown to be promising activators of sirtuins [38, 52].

### 5.2. Mechanisms of Autophagy Induction by Oleuropein Aglycone

One of the polyphenols better characterized as an autophagy inducer is oleuropein aglycone (OLE), which is found in EVOO. Oleuropein is a secoiridoid glycoside with a phenylpropanoid alcohol obtained from the biosynthesis of mevalonic acid. From the chemical point of view, it is an 11-methyl-ester (elenolic acid glucoside) ester combined with 3,4-dihydroxyphenylethanol (3,4-DHPEA). It is the major phenolic component of the pulp of the green olives and leaves of the *Gentianaceae*, *Cornaceae*, and *Oleaceae* families, in the latter family being especially relevant in the variety *Olea europaea* L. (which gives its name to the glycoside) [58]. After the process of maturation and extraction of the olive juice, in the EVOO, due to the action of the enzyme *β*-glucosidase, we can only find this secoiridoid in its aglyconic form as OLE (3,4-DHPEA-EA), being aldehydic form of OLE, the main responsible for its bitter taste [59, 60]. The relative amount of OLE in EVOO depends on the variety of olive fruit used, being in many of them one of the most abundant polyphenols [61].

Several in vitro and in vivo studies have demonstrated the multiple benefits of oleuropein and its derivatives associated to their antioxidant, antidiabetic, antimicrobial, antiviral, antitumor, hepatoprotective, cardioprotective, neuroprotective, antiaging, and anti-inflammatory properties [62–65]. In terms of neuroprotective activity, the most recent experimental studies have shown that OLE reduces cognitive impairment and improves synaptic function in animal models. This is due to the inhibition of the aggregation and toxicity of Tau [66] and A*β* [67], the epigenetic modulation by histone acetylation [68], the reduction of astrocytosis and modulation of astroglia activity, and the induction of autophagy [69].

The effects of OLE on Tau aggregation was demonstrated by studying the antiaggregant capacity of oleuropein and its derivatives (OLE and hydroxytyrosol) over wild-type and P301L Tau protein in vitro. They were able to obtain results similar to methylene blue in inhibiting fibrillization at low micromolar concentrations of Tau, demonstrating a higher inhibitory capacity of OLE related to the presence of aldehyde groups in its structure [66]. A posterior study demonstrated the inhibition of A*β* aggregation using the transgenic strain CL2006 of *Caenorhabditis elegans*, a simplified model of AD expressing human A*β* peptide in the cytoplasm of muscle cells of the body wall. They found that larvae fed with OLE showed a reduction of A*β* plaque deposits, a lower content of toxic A*β* oligomers, a marked decrease of paralysis, and an increase of life expectancy with respect to untreated animals [67].

On the other hand, several reports have associated the effect of OLE to a positive modulation of autophagy pathway that leads to cognitive improvement in animal models. One of the first works that demonstrated this hypothesis was the study carried out by Grossi and collaborators using wild-type and TgCRND8 transgenic mice, as mentioned before, a model for human A*β* pathology [35, 70]. In this study, mice whose diet was supplemented with OLE (50 mg/kg of food) exhibited an increase of autophagic vesicles. This was demonstrated by the augmented levels of Beclin-1 and LC3 in the soma and dendrites of neurons from different parts of the somatosensory/parietal and entorhinal/piriform cerebral cortex correlating with increased LC3II/LC3I ratio. This induction was more significant in TgCRND8 transgenic mice rather than in wild-type. Additionally, these authors demonstrated that OLE improved the autophagosome-lysosome fusion measured as the increase of p62 and cathepsin B levels in OLE supplemented TgCRND8 mice up to the levels found in wild-type mice. They also reported the colocalization of both p62 and cathepsin B labels suggesting a proper fusion of lysosomes to autophagic vesicles and, therefore, functional degradation phase of autophagy. They proposed that the mechanism of autophagy activation might be due to the inhibition of mTOR pathway reflected in the decrease of phosphorylation of its target p70S6 protein kinase in cell culture.

#### 5.2.1. OLE as Modulator of Ca^+2^-CaMKK-AMPK-mTOR Axis

**Figure 2:**
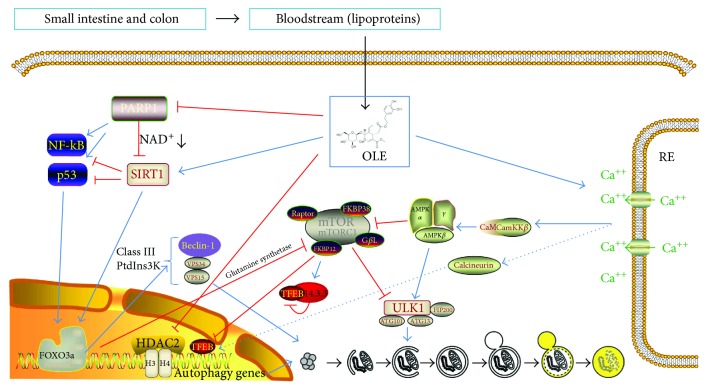
Pleiotropic action of OLE over autophagy induction. Summary of autophagy pathways in which OLE has demonstrated an effect.

Initially, it was proposed that the mechanism by which OLE induced autophagy would be through the increase of cytosolic levels of Ca^+2^ and the subsequent activation of the enzyme complex AMPK through Ca^2+/^Calmodulin Protein Kinase Kinase *β* (CaMKK*β*). This complex facilitates mTORC1 inhibition and ULK1 activation to generate autophagic vacuole induction [71] (Figure 2). This is also the case of other polyphenols such as resveratrol [72] and epigallocatechin gallate (EGCG) [73]. Accordingly, they demonstrated a biphasic phosphorylation of the regulatory residue Thr172 of AMPK correlating with elevated Beclin-1 levels in SH-SY5Y cells treated with 50 *μ*M OLE [71]. This was mediated by the biphasic increase of intracellular Ca^2+^ levels that come from the endoplasmic reticulum that induce CaMKK*β* activation. This correlates with a fast increase of Beclin-1 levels that was proposed to arise from the Beclin-1 fraction complexed with Bcl-2/Bcl-xL in the cytoplasm rather than to new synthesis. The release of Beclin-1 from this complex is critical for inducing autophagy because free Beclin-1 interaction with VPS34 is needed to initiate autophagosome formation [74]. The activation of autophagy by OLE was partially inhibited by STO-609 and component C, inhibitors of CaMKK*β* and AMPK, respectively, suggesting that autophagy activation by OLE occurs mainly through the Ca^2+^ increase that induces CaMKK*β* activation and the subsequent AMPK phosphorylation [71].

All these results together indicate that OLE activates AMPK, which can allow the formation of ULK1 quaternary complex directly or indirectly by the inhibition of mTOR that inhibits ULK1 (Figure 2). ULK1 promotes autophagy by Beclin-1 phosphorylation and VPS34 lipid kinase activation that produces phosphatidylinositol 3-phosphate, necessary for the formation of the early autophagosomal membrane [75].

#### 5.2.2. OLE as Modulator of PARP1-SIRT1 Axis

Multiple polyphenols are known to induce autophagy by the activation of sirtuins (SIRT) [52]. SIRT1 deacetylates many transcription factors such as p53, NF-*κ*B, and FOXO, a mediator of autophagy. SIRT1 could influence autophagy directly via deacetylation of key components of the autophagy induction network, such as the products of autophagy genes Atg5, Atg7, and Atg8 [76]. The treatment of TgCRND8 mice with OLE (50 mg/Kg of diet) was able to reduce the activation of Poly (ADP-ribose) polymerase-1 (PARP1) at both RNA and protein levels as well as the subsequent accumulation of PAR polymers up to the levels found in wild-type mice [77]. Moreover, OLE was able to abolish the increase of the apoptotic mediators phospho-NF-*κ*B and phospho-p53 Ser46 in these animals. PARP1 activation causes a reduction of NAD^+^ levels that result in SIRT1 inhibition [78]. Therefore, OLE-mediated reduction of PARP1 increased NAD^+^ levels that were able to induce SIRT1 in TgCRND8 mice [77] (Figure 2). Accordingly, the treatment of N2a cells with OLE 100 *μ*M for 24 h was able to reverse the PARP1 activation caused by methylnitronitrosoguanidine (MNNG), a mutagen that activates PARP1 expression, as well as increased SIRT1 and Beclin-1 [77].

#### 5.2.3. OLE as Epigenetic Modulator

It is also worth mentioning the action of OLE as an epigenetic modulator. It is known that abnormal acetylation takes place in memory and learning disorders such as AD, where significant increase of histone deacetylase 2 (HDAC2) inhibits gene expression of specific locus, such as autophagy markers [79]. Moreover, histone acetylation has been shown to ameliorate cognitive deficits in AD animal models suggesting its targeting as a promising therapeutic strategy for this disease [79]. Polyphenols have shown to regulate gene expression by modulating histone acetylation and DNA methylation, as is the case of EGCG in cancer cells [80].

Noteworthy, TgCRND8 mice showed increased levels of HDAC2 correlating with decreased levels of histone 3 acetylation on lysine 9 (H3K9) and of histone 4 acetylation on lysine 5 (H4K5) in cortex and hippocampus [68]. The treatment with OLE (50 mg/kg of diet for 8 weeks) significantly decreased the levels of HDAC2 in both wild-type and TgCRND8 mice and increased H3K9 and H4K5 especially in the transgenic model (Figure 2). This was accompanied by an improvement of synaptic function revealed by restoring high-frequency stimulation-induced long-term potentiation as well as 3-theta burst and high-frequency stimulation-evoked posttetanic potentiation in slices of brains of OLE-treated transgenic mice.

On the other hand, transcription factor EB (TFEB) is a master regulator of lysosomal and autophagic function [81, 82]. Previous studies demonstrated that mTOR-mediated phosphorylation of TFEB in Ser 211 promotes the interaction of TFEB with the 14-3-3 protein and results in a cytoplasmic localization, therefore inhibiting its function as a transcriptional factor [83] (Figure 2). It has been recently shown that activation of calcineurin by lysosomal Ca^2+^ releases binds and dephosphorylates TFEB, thus promoting its nuclear translocation inducing autophagy and lysosomal biogenesis [84]. As we previously mentioned, OLE was able to increase intracellular Ca^2+^ that activates Ca^2+^/CaMKK*β*/AMPK axis [71]. Hence, Ca^2+^ release coming from different organelles might induce autophagic flux independently but possibly, synergistically. Further studies will be necessary to unravel if intracellular Ca^2+^ increase mediated by OLE is able to activate calcineurin and the subsequent TFEB dephosphorylation and activation which induce autophagy (Figure 2).

## 6. Bioavailability and Effective Dose of OLE

Before presuming an effect of OLE in neurons in humans, it is important to know its bioavailability in the organism once ingested and if it arrives in its full form or with modifications. In humans, it has been demonstrated that the apparent absorption of olive oil phenols, among which is OLE, ranges between 55 and 66% of the ingested dose [85]. This is performed mainly in the small intestine and in a minor proportion in the colon and, once in the blood, the oleuropein is transported by lipoproteins where it is rapidly distributed [85, 86] (Figure 2). The mechanism of OLE absorption is still unknown, as well as those of other olive oil polyphenols [87]. However, it is known that intestinal absorption and renal clearance of oleuropein and hydroxytyrosol are relatively rapid, reaching their maximum plasma concentration at half an hour after ingestion, followed by a rapid decline culminating in 2.5 hours [86]. The main metabolites that can be found in plasma and urine after oleuropein ingestion are hydroxytyrosol and its conjugates (sulfated and glucuronidated) followed by OLE [86]. In addition, there is a strong individual component regarding the bioavailability and metabolism of oleuropein depending on the gender, being men the most efficient in conjugating this substance, which explains the lower plasma levels compared to the increased levels of conjugated forms of hydroxytyrosol [86]. On the other hand, the absorption of oleuropein is much more efficient if the compound is taken in liquid form rather than ingestion in capsules [86].

In addition, it has been suggested that OLE, being one of the less polar compounds of the olive oil polyphenols, is mostly transformed in hydroxytyrosol either in the gastrointestinal tract before it is absorbed or in the intestinal cells, blood, or liver after its absorption [85]. Nonetheless, it has been recently demonstrated that hydroxytyrosol is able to induce autophagy in chondrocytes after oxidative stress exposure mainly, but not exclusively, by SIRT1 induction [88]. This opens the possibility that not only OLE but also its derivatives are able to induce the protective autophagy induction described in this review.

The autophagy induction by OLE can occur even at low doses. It was demonstrated that high concentrations (50 mg/kg) of a mixture of polyphenols present in olive mill waste water (among which was OLE), as well as medium (12.5 mg/kg) and low concentrations (0.5 mg/kg) of OLE increased autophagic activity in the cortex of TgCRND8 mice [89]. They could also observe that 12.5 mg/kg of OLE or 50 mg/kg of polyphenols mix significantly improved cognitive functions and diminished A*β* deposition of TgCRND8 mice.

Considering a variety of EVOO specially enriched in OLE as is Seggianese oil, an Italian olive oil whose OLE content is above 30% of total secoiridoids of which are ranging 619 ± 128 mg/L [90], the amount of OLE is over 185.7 mg/L in this EVOO variety. The daily recommendation of EVOO consumption in the Mediterranean diet is 25–50 ml [91] that would represent between 4.6 and 9.3 mg of OLE using Seggianese oil. Taking into consideration the differences of weight between mice (20 g) and humans (e.g., 60 Kg), the daily concentration of OLE would be the equivalent to 1.5–3.1 *μ*g/day in mice. The OLE daily intake at which autophagy induction was achieved in the study by Pantano et al. was 0.5 mg/kg of food [89]. Considering that mice model eats 3–5 g/day (http://www.researchdiets.com), this would correspond to a dose of 1.5–2.5 *μ*g/day of OLE. As we calculated before, this amount of OLE was equivalent to the daily intake of Seggianese oil. The minimum dose in the study by Pantano et al. that achieved functional recovery and reduced amyloid beta burden was 12.5 mg/Kg of food [89] that would correspond to 37.5–62.5 *μ*g/day of OLE. This amount exceeds the OLE quantity guaranteed by the daily intake of EVOO and this may indicate that the effect of EVOO might not be enough in advanced stages of the disease. However, EVOO contains many other components with potential beneficial effects over health and cognitive improvement apart from OLE. Therefore, further studies are necessary to determine the minimum active dose of OLE and the potential benefit of EVOO intake.

In addition, administration of 50 mg/Kg OLE in the diet was safe and none of the TgCRND8 mice involved in the experiment died or suffered any side effects due to high intake of OLE [70]. Moreover, the presence of several foods with substantial levels of polyphenols is one of the main explanations for the healthy properties of the Mediterranean and Asian diets [92, 93]. Taking in consideration all these studies, we can deduce that high doses of polyphenols might not be harmful to humans. However, specific studies of OLE intake in humans are necessary to rule out its possible toxicity.

Many open questions remain regarding the action of OLE once ingested and whether it is able to reach the neurons and exert its function. Furthermore, the determination of these metabolites' bioactivity and the levels at which OLE can become toxic needs to be analyzed. Regarding the possible effect of OLE in humans, further studies are necessary to determine OLE stability in human gastric fluids and blood. Additionally, it is important to unravel whether OLE is absorbed and is able to cross the blood-brain barrier unmodified. One important limitation for these studies is that purified OLE is not available for human consumption so far. Notwithstanding, there are many ongoing studies that analyze the beneficial effects of EVOO consumption in humans; from those, it might be possible to extrapolate conclusions about OLE effect. With this aim, it will be necessary to determine the exact concentration of OLE in a concrete EVOO variety and supplement volunteers with an exact daily dose. This would allow to estimate the concentration of OLE and its derivatives in blood and cerebrospinal fluid. However, being EVOO a mixture of many polyphenols, which most of them generate similar derivatives, this determination might be inexact. Moreover, as several polyphenols and derivatives may have similar beneficial effects on health, it would be difficult to attribute this outcome to one single compound. It is mandatory to clarify if the autophagy induction mediated by OLE depends on its unmodified form or on its conversion in hydroxytyrosol. This issue would be easier to achieve in vitro by studying autophagy induction after treatment of human cells, such as SH-SY5Y, with equivalent doses of OLE or hydroxytyrosol and evaluating their stability during experimental conditions.

Finally, unless we could acquire purified OLE for human consumption, the conclusions obtained from these studies will be merely speculative and should be carefully supported by equivalent experiments in animals where purified OLE and EVOO can be compared.

In summary, the cognitive improvement developed in animal models of Alzheimer's disease, such as TgCRND8 mice [70], indicates that supplementation of the diet with OLE may have beneficial effects in slowing cognitive decline in these patients. This clearly indicates that either directly OLE or its derivatives are able to cross the blood-brain barrier and develop their neuroprotective function in the brain, where a decrease in protein aggregates and a significant activation of autophagy were observed.

## 7. Conclusions

Polyphenols are known to be the substances responsible for the neuroprotective properties attributed to the Asian and Mediterranean diets, rich in foods that contain a large amount of these compounds of plant origin [50]. We have focused our attention in OLE, one of the polyphenols abundant in EVOO and one of the bases of the Mediterranean diet [94].

In the present work, we have summarized all the works that have demonstrated that OLE reduced symptoms of AD and cognitive impairment [68, 70, 71, 89]. Several studies have proposed that OLE mechanism of action associated to this cognitive improvement was by autophagy induction that has been also shown to reduce amyloid aggregates [50, 68–71, 77, 89]. Furthermore, OLE does not show side effect (cell death or apoptosis), as is the case of some polyphenols such as curcumin [48], nor neurodegeneration as is the case of prolonged treatment with rapamycin [32]. This, together with its ability to fight cytotoxicity derived from the accumulation of A*β* and reduce inflammation derived from the activation of astrocytes and microglia are responsible for the decrease in cognitive impairment in TgCRND8 mice. Unfortunately, there are no evidences of OLE benefits in humans due to the complexity of these studies. But extrapolating the results of the studies that related adherence to the Mediterranean diet [40] or olive oil consumption [42] with the decrease in the prevalence of neurodegenerative diseases, the cognitive improvement in AD mice model [70] and the results in human SH-SY5Y neuroblastoma cells [71], we can conclude that OLE consumption might be useful for delaying cognitive impairment in humans.

The data presented in our study confirm that OLE is a compound capable of inducing autophagy in both in vitro and in vivo models and that this leads to an improvement in cognitive impairment as well as in *β* amyloid and Tau aggregation. Therefore, based on studies on the consumption of EVOO and adherence to the Mediterranean diet, rich in polyphenols including OLE, we can hypothesize OLE would be useful to prevent and lessen symptoms associated with AD. However, more studies are needed to test the effects of OLE in humans, in terms of metabolic pathways and bioavailability, as well as to demonstrate the effects of OLE on gene expression.
